# Proteoform-Resolved FcɤRIIIa Binding Assay for Fab Glycosylated Monoclonal Antibodies Achieved by Affinity Chromatography Mass Spectrometry of Fc Moieties

**DOI:** 10.3389/fchem.2019.00698

**Published:** 2019-10-24

**Authors:** Steffen Lippold, Simone Nicolardi, Manfred Wuhrer, David Falck

**Affiliations:** Center for Proteomics and Metabolomics, Leiden University Medical Center, Leiden, Netherlands

**Keywords:** affinity chromatography, mass spectrometry, middle-up protein analysis, cetuximab, Fc glycosylation, Fab glycosylation, FcɤRIIIa, Kgp

## Abstract

Fcɤ receptors (FcɤR) mediate key functions in immunological responses. For instance, FcɤRIIIa is involved in antibody-dependent cell-mediated cytotoxicity (ADCC). FcɤRIIIa interacts with the fragment crystallizable (Fc) of immunoglobulin G (IgG). This interaction is known to be highly dependent on IgG Fc glycosylation. Thus, the impact of glycosylation features on this interaction has been investigated in several studies by numerous analytical and biochemical techniques. FcɤRIIIa affinity chromatography (AC) hyphenated to mass spectrometry (MS) is a powerful tool to address co-occurring Fc glycosylation heterogeneity of monoclonal antibodies (mAbs). However, MS analysis of mAbs at the intact level may provide limited proteoform resolution, for example, when additional heterogeneity is present, such as antigen-binding fragment (Fab) glycosylation. Therefore, we investigated middle-up approaches to remove the Fab and performed AC-MS on the IgG Fc to evaluate its utility for FcɤRIIIa affinity assessment compared to intact IgG analysis. We found the protease Kgp to be particularly suitable for a middle-up FcɤRIIIa AC-MS workflow as demonstrated for the Fab glycosylated cetuximab. The complexity of the mass spectra of Kgp digested cetuximab was significantly reduced compared to the intact level while affinity was fully retained. This enabled a reliable assignment and relative quantitation of Fc glycoforms in FcɤRIIIa AC-MS. In conclusion, our workflow allows a functional separation of differentially glycosylated IgG Fc. Consequently, applicability of FcɤRIIIa AC-MS is extended to Fab glycosylated IgG, i.e., cetuximab, by significantly reducing ambiguities in glycoform assignment vs. intact analysis.

## Introduction

**Figure 1:**
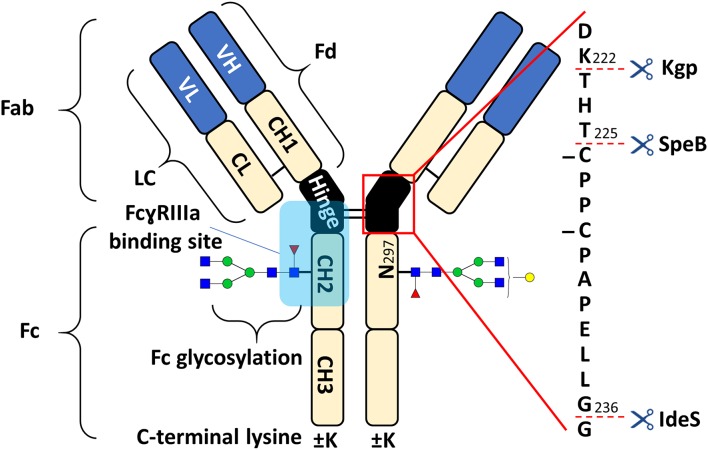
Schematic overview of human IgG1 with a zoom into the hinge region, indicating cleavage sites of IdeS, SpeB, and Kgp. The heavy chain (HC) contains three constant domains (CH1–CH3) and a variable domain (VH), whereas the light chain (LC) has only one constant domain (CL) and a variable domain (VL). The Fd consists of VH and CH1. LC and Fd together form the antigen binding fragment (Fab). About 15% of plasma IgG contain one or more additional *N*-glycosylation sites in the variable domains (Anumula, 2012). CH2 and CH3 of the two heavy chains build the fragment crystallizable (Fc). Within the Fc is a conserved glycosylation site at N_297_ in the CH2 (Jefferis, 2009). Between the CH1 and CH2 of the heavy chains is a mostly flexible hinge region and the four chains are covalently connected via disulfide bridges. Amino acids are presented with single letter code and numbered according to the Kabat system (Kabat et al., 1991).

The fragment crystallizable (Fc) of antibodies mediates immunological responses, for example through binding to Fc receptors (Nimmerjahn and Ravetch, 2008; Pincetic et al., 2014). Fc glycosylation has a key role in modulating Fc receptor-mediated effector functions, such as antibody-dependent cell-mediated cytotoxicity (ADCC) (Reusch and Tejada, 2015; Cymer et al., 2018; Saunders, 2019). The affinity toward FcɤRIIIa is known to be crucial for ADCC (Nimmerjahn and Ravetch, 2008). Fucosylation of Fc glycans drastically decreases FcɤRIIIa affinity which is attributable to an unique glycan-glycan interaction (Ferrara et al., 2011). Other glycosylation features such as galactosylation were also shown to affect the Fc-FcɤRIIIa interaction (Thomann et al., 2015; Dekkers et al., 2017). The binding of the Fc to FcɤRIIIa is asymmetric in a 1:1 stoichiometry (Sondermann et al., 2000). Nonetheless, FcɤRIIIa affinity is influenced by the pairing of Fc glycans (Shatz et al., 2013). While differential affinity of glycoforms is dominated by the stronger binding glycan, the second glycan modulates affinity to a smaller extent, but along the same structural features (Shatz et al., 2013; Lippold et al., 2019). Nowadays, therapeutic monoclonal antibodies (mAbs) are most often derived from human immunoglobulin G 1 (IgG1, schematic overview in Figure 1). They are used in the treatment of various diseases, such as cancers or autoimmune diseases (Chan and Carter, 2010; Weiner et al., 2010). In the biopharmaceutical industry, mAbs are very successful and currently dominate new approvals (Walsh, 2018). Recently, glycoengineering for enhanced FcɤRIIIa affinity and ADCC has been therapeutically exploited (Jefferis, 2009; Beck and Reichert, 2012).

Numerous analytical technologies exist for assessing the effector functions of therapeutic antibodies (Jiang et al., 2011; Cymer et al., 2018). They vary largely in information content, generally with a negative correlation between complexity and resolution. Complex cellular assays are more easily translated to the *in vivo* situation. Contrary, physicochemical assays provide higher molecular resolution and better robustness. Though immune responses depend on the formation of immune complexes, receptor binding studies on monomeric IgG are highly relevant and widely used (Nimmerjahn and Ravetch, 2008; Cymer et al., 2018). Ultimately, combining information from different assays is essential to fully understand antibody effector functions. Glycosylation heterogeneity is a major challenge for the assessment of individual contributions of specific glycoforms to the effector functions, especially considering pairing possibilities. Several studies applied laborious glycoengineering in order to assess receptor binding and effector functions of specific glycoforms (Dashivets et al., 2015; Thomann et al., 2015; Dekkers et al., 2017; Wada et al., 2019). Affinity chromatography (AC) represents a cell-free physicochemical assay which provides a functional separation and correlates well with surface plasmon resonance (SPR) assays and ADCC assays (Dashivets et al., 2015; Thomann et al., 2015; Wada et al., 2019). We reported recently on coupling of FcɤRIIIa AC to mass spectrometry (AC-MS) (Lippold et al., 2019). This approach allows the differential assessment of Fc glycoforms in heterogeneously glycosylated mAbs with high resolution of proteoforms and affinity on an intact protein level. Whereas it should be very powerful for most mAbs, proteoform resolution may be insufficient for more complex formats (Ayoub et al., 2013). This applies to mAbs with a higher degree of heterogeneity due to sequence variants or post translational modifications (PTMs), especially additional glycosylation sites in the antigen-binding fragment (Fab). In addition, the analysis of new antibody-derived therapeutic formats, such as bispecific antibodies or fusion proteins, may be challenging (Klein et al., 2016).

Cetuximab is an approved mAb with additional Fab glycosylation and ADCC is described as one mechanisms of action (Kurai et al., 2007; Kol et al., 2017). Each heavy chain (HC) contains an *N*-glycosylation site at the Fab (N_88_) and at the Fc (N_299_) resulting in a high number of possible glycoforms. The proteoform heterogeneity of cetuximab is further increased by C-terminal lysine variants of the HC (Ayoub et al., 2013). Hence, glycoform assignment of the heavily glycosylated cetuximab by intact mass analysis is hindered by a high degree of ambiguities (Ayoub et al., 2013; Bern et al., 2018). Middle-up approaches are highly advantageous alternatives for obtaining information about individual subunit (e.g., Fc, Fc/2, Fab) modifications, especially for complex formats (Beck et al., 2013; Sjögren et al., 2016; Lermyte et al., 2019). Bacterial enzymes are important tools for middle-up approaches, since they cleave IgG specifically within the hinge region. Robust and simple workflows for the middle-up analysis of (therapeutic) mAbs are established (Zhang et al., 2016; Moelleken et al., 2017; Sjögren et al., 2017; Bern et al., 2018; van der Burgt et al., 2019). IdeS, SpeB, and Kgp are frequently used commercial IgG hinge-specific proteases (cleavage sites and products are indicated in Figure 1 and Supplementary Figure 1, respectively). As opposed to papain, for example, additional cleavages outside of the hinge region are not reported under standard conditions. Their characteristics were recently summarized (Sjögren et al., 2017). IdeS based middle-up MS analysis of cetuximab is commonly applied to unravel the (glycosylation) microheterogeneity (Ayoub et al., 2013; Janin-Bussat et al., 2013; Bern et al., 2018).

This study combines our recently reported FcɤRIIIa AC-MS with middle-up analysis. Therefore, we investigated how cleavages within the hinge region affect the FcɤRIIIa binding properties of the obtained Fc. Three different commercial IgG hinge-specific proteases were tested, namely IdeS, SpeB, and Kgp (Sjögren et al., 2017). We demonstrate comparability of middle-up and intact affinity assessment by FcɤRIIIa AC-MS upon Kgp digestion. Furthermore, we applied this workflow to cetuximab and simultaneously assessed the FcɤRIIIa affinity, characterized the Fc glycoform pairings and analyzed the Fab glycosylation.

## Materials and Methods

### Chemicals, Proteases, and Antibodies

All chemicals in this study had at least analytical grade quality. Deionized water was obtained from a Purelab ultra (ELGA Labwater, Ede, The Netherlands). Preparation of mobile phase was performed with ammonium acetate solution (7.5 M, Sigma-Aldrich, Steinheim Germany) and glacial acetic acid (Honeywell, Seelze, Germany). IdeS (FabRICATOR®), SpeB (FabULOUS®), and Kgp (GingisKHAN®) proteases were purchased from Genovis (Lund, Sweden). A reference standard therapeutic mAb produced in CHO cells (referred to as mAb1) and the FcɤRIIIa affinity column was obtained from Roche Diagnostics (Penzberg, Germany). An EMA-approved cetuximab (Erbitux®) was used in this study. Cetuximab is a chimeric IgG1, produced by SP2/0 murine myeloma cells, which binds to the epidermal growth factor receptor (EGFR).

### Antibody Digestion (IdeS, SpeB, Kgp)

All IgG hinge-specific proteases were reconstituted in deionized water following the manufacturer's instructions (IdeS: 67 units/μL, SpeB 40 units/μL, Kgp: 10 units/μL). Buffers and reducing conditions were selected from the recommended options. mAbs were digested at a concentration of 5 mg/mL (1 unit of protease per 1 μg of mAb) and incubated for 1 h at 37°C. In case of Kgp, the samples were buffer exchanged prior to digestion (10 kDa molecular weight cut-off filter, Merck, Darmstadt, Germany) to digestion buffer. 100 mM Tris buffer (pH 8) was used with mild reducing conditions (2 mM cysteine) for Kgp and reducing conditions (1 mM DTT) for SpeB, respectively. IdeS digestion was performed under non-reducing conditions with 100 mM ammonium bicarbonate (pH 7). After digestion, samples were buffer exchanged to a final concentration of 5 mg/mL in 50 mM ammonium acetate pH 5 (10 kDa molecular weight cut-off filter).

### FcɤRIIIa Affinity Chromatography—Mass Spectrometry

The FcɤRIIIa AC-MS system was previously described in detail (Lippold et al., 2019). In short, a biocompatible Thermo Ultimate3000 instrument coupled to a 15 T solariX XR FT-ICR mass spectrometer (Bruker Daltonics, Bremen, Germany) was used. The column was operated at 25°C and a flow rate of 500 μL/min was applied. Prior to MS detection, the flow rate was reduced to 30 μL/min via flow-splitting. Mobile phase A was 50 mM ammonium acetate pH 5 and mobile phase B 50 mM ammonium acetate pH 3. Upon injection (50–100 μg sample), the column was washed for 10.5 min (5 column volumes) with 100% mobile phase A and then a linear gradient to 42 min to 100% mobile phase B (15 column volumes) was applied. For electrospray ionization (ESI), the capillary voltage was set to 4,000 V, the nebulizer gas to 0.8 bar, the dry gas flow to 3 L/min dry gas and the source temperature to 200°C.

### Data Analysis

Average masses were calculated using the web-based Protein Tool (https://www.protpi.ch) based on the protein sequences and expected modifications. For mAb1 and cetuximab, C-terminal lysine clipping and 16 disulfide bridges were set as modifications. N-terminal pyroglutamic acid was additionally added as modification for cetuximab. Visualization and processing of mass spectra was performed in DataAnalysis 5.0 (Bruker Daltonics). Extracted ion chromatograms (EICs) were generated based on the theoretical *m/z* (±0.2 Th) for all observed charge states. For deconvolution, the Maximum Entropy tool was used (deconvolution range indicated in table headings, data point spacing = 1, instrument resolving power = 3,000). All described Fc glycans can be found in Supplementary Table 1 which provides information about composition and structure.

## Results and Discussion

### IgG Protease Evaluation

**Figure 2:**
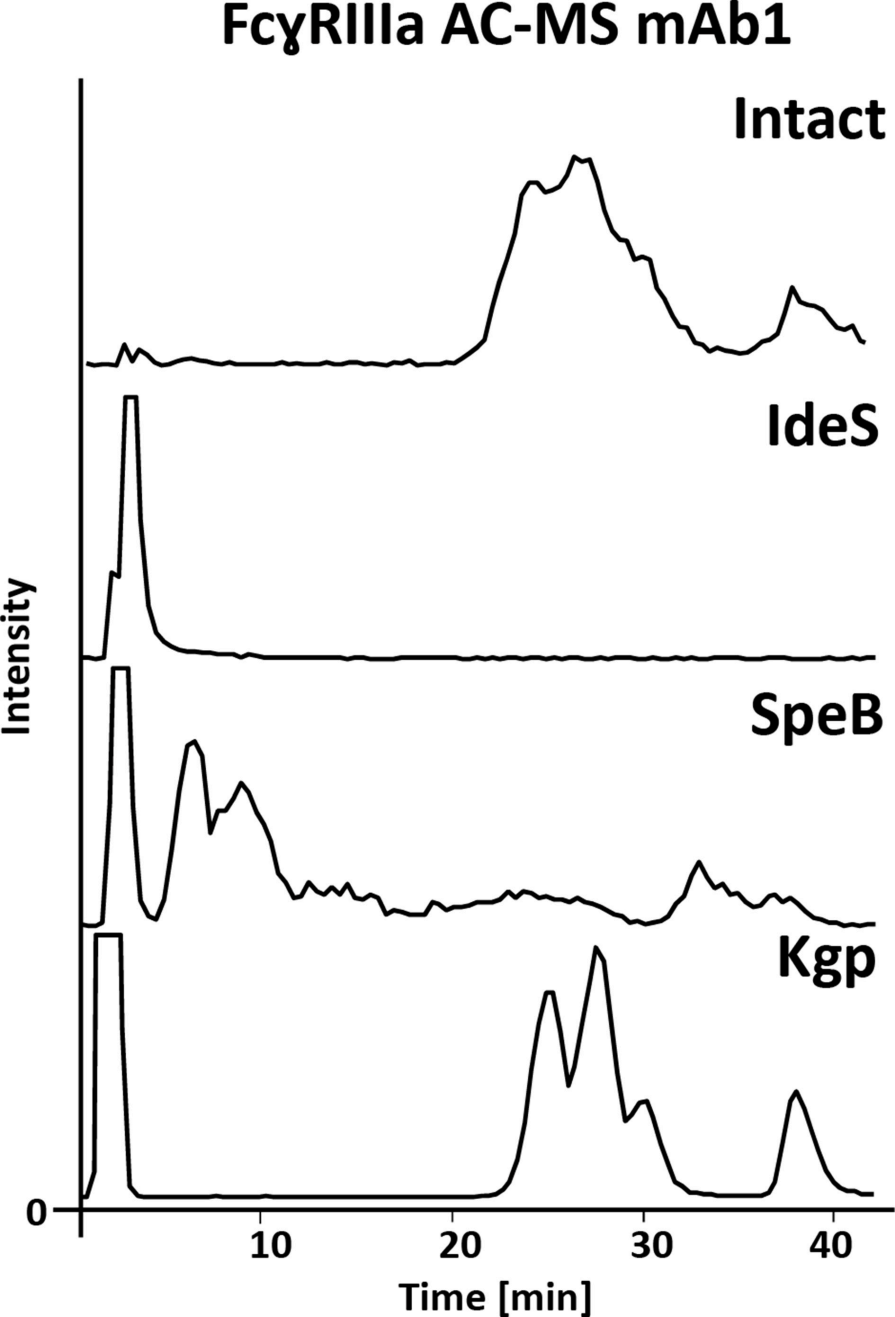
FcɤRIIIa AC-MS base peak chromatograms of intact and IdeS-, SpeB-, and Kgp-digested mAb1. For intact and Kgp-digested mAb1, two distinct groups of peaks were detected, namely fucosylated forms with lower affinity (RT 20–35 min) and afucosylated glycoforms with higher affinity (RT higher than 35 min); see Figure 3.

The FcɤRIIIa AC-MS retention profiles of hinge cleaved mAb1, obtained by either IdeS, SpeB, or Kgp, and of intact mAb1 were compared (Figure 2). Although digestion sites of the three proteases are in close proximity in the hinge region (Figure 1), vastly different retention profiles were observed for the differently cleaved Fc. Kgp generated Fc was found to exhibit a remarkably comparable retention profile to the intact mAb1. IdeS digested mAb1 did not show retention on the FcɤRIIIa column and the expected cleavage products, including the Fc, were detected in the injection peak (Supplementary Figure 2). Under native conditions, Fc fragments consisting of paired polypeptide chains were observed rather than single Fc/2 chains which is attributable to non-covalent interactions of the Fc polypeptides (Bern et al., 2018). The lack of retention can be explained by the removal of amino acids that form an essential part of the FcɤRIIIa binding motif (Sondermann et al., 2000). In particular, L_234_ and L_235_ are crucial amino acids. The mutation of these amino acids to alanines (LALA mutant) is known to eliminate FcɤRIIIa binding and thus ADCC (Schlothauer et al., 2016; Saunders, 2019). In contrast to IdeS, the protease SpeB does not remove these key amino acids from the Fc. The Fab was observed in the injection peak while the Fc was retained on the FcɤRIIIa column (Supplementary Figure 3). However, in contrast to Kgp, the Fc retention profile upon SpeB cleavage was vastly different from that of the intact mAb. SpeB derived Fc spread over the entire chromatogram and most of the Fc eluted already before the pH gradient started. Two differences from Kgp derived Fc might provide an explanation. Firstly, the removed amino acids (THT) might lead to an impaired conformational stability of the Fc obtained by SpeB. Furthermore, substitution of H, at this position, was shown to influence FcɤRIIIa binding and ADCC (Yan et al., 2012). Finally, partial reduction, which is likely to occur under the applied reducing conditions of 1 mM DTT (Sjögren et al., 2017), is discussed to influence binding to Fc receptors and ADCC (Liu and May, 2012). Under milder reducing conditions SpeB does not show sufficient activity (data not shown). Besides the impaired retention profile, an increased heterogeneity is a disadvantage for MS analysis, when comparing SpeB to Kgp. Heterogeneity is caused by additional cleavages between H_224_ and T_225_ or T_223_ and H_224_ (Supplementary Figure 3). These products showed a similar impaired binding behavior. In contrast to SpeB, Kgp protease retains T_223_, H_224_, and T_225_ and works under mild reducing conditions (2 mM cysteine) which prevents reduction of the hinge interchain disulfide bonds (Moelleken et al., 2017). Both, the additional amino acids and intact disulfide bonds, might be responsible for improved binding of Kgp derived Fc over SpeB derived Fc. Interestingly, the Fab was removed in a previous study using papain to exclude that the Fab influences the FcɤRIIIa binding (Dashivets et al., 2015). In line with our observations for Kgp in FcɤRIIIa AC-MS, binding of papain generated Fc was comparable to intact IgG (glycovariants). Total binding strength as well as glycoform differences were preserved in the SPR analysis (Dashivets et al., 2015). Papain digestion is performed under conditions (5–10 mM cysteine) which are only slightly more reductive than for Kgp (2 mM cysteine) (Dashivets et al., 2015; Moelleken et al., 2017). The preferred cleavage site of papain is between the Kgp and SpeB cleavage site (H_224_ and T_225_) (Kim et al., 2016; van den Bremer et al., 2017) and corresponds to the observed additional cleavage site of SpeB. Hence, the harsher incubation conditions are more likely to cause the differences between Kgp, or papain, and SpeB than the presence or absence of H_224_. However, by-products (due to cleavage outside the hinge region), insufficient yields and glycoform dependency make papain less favorable as an IgG middle-up protease (Raju and Scallon, 2007; Moelleken et al., 2017). Moreover, papain might degrade the receptor material of the affinity column, since it is not an IgG-specific protease. Consequently, due to the preservation of the affinity separation and the specificity, Kgp was chosen for the middle-up FcɤRIIIa AC-MS workflow. Interestingly, an IdeS middle-up approach is described for neonatal Fc receptor (FcRn) AC (Schlothauer et al., 2013). In this study, an impact of the Fab on the FcRn interaction was shown for several mAbs. The influence of different Fabs might also be relevant for other Fc receptor interactions. Thus, middle-up AC-MS also has high potential for investigating Fab-Fc structure-function relationships in a proteoform-resolved manner.

### Comparability of Intact and Middle-Up FcɤRIIIa Affinity Chromatography

**Figure 3:**
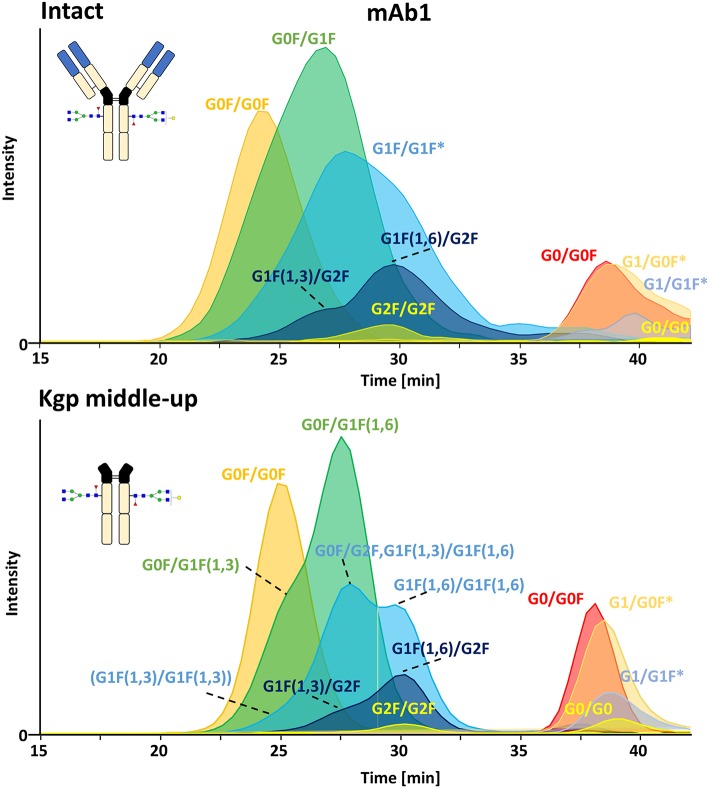
Extracted ion chromatograms of major glycoforms assigned for FcɤRIIIa AC-MS of mAb1 on intact and Kgp middle-up level. *indicates additional isomers listed in Supplementary Table 2.

FcɤRIIIa AC, separates two distinct groups of peaks, representing fucosylated glycoforms (2x fucosylated glycoforms) with lower affinity and (partially) afucosylated glycoforms (2x and 1x afucosylated glycoforms) with higher affinity (Figures 2, 3) (Lippold et al., 2019). It has to be noticed that the column performance was slightly different for late eluting glycoforms compared to previous experiments on intact mAb1 (Lippold et al., 2019). Fc glycoforms are discussed in a nomenclature as listed in Supplementary Table 1. Middle-up FcɤRIIIa AC-MS exhibited sharper peaks than its intact counterpart (Figure 3). In general, decreasing the size of proteins in chromatography, in this case from 150 to 50 kDa, increases the diffusion coefficient (Tyn and Gusek, 1990). This improves the mass transfer kinetics of the protein (Gritti and Guiochon, 2012; Astefanei et al., 2017). Sharper peaks and a similar retention resulted in better separation efficiency for the middle-up FcɤRIIIa AC-MS. Mainly, several partially separated species in the EICs of Figure 3 indicate separation of glycoforms with terminal galactose present on the 1,3-arm or the 1,6-arm of G1F. Differential FcɤRIIIa affinity of G1F(1,3) and G1F(1,6) glycoforms has recently been reported [G2F = G1F(1,6) > G1F(1,3) = G0F] (Aoyama et al., 2019). Based on this, G0F/G1F(1,3) has a similar affinity as G0F/G0F. G0F/G1F(1,6) exhibits an increased FcɤRIIIa affinity comparable to G0F/G2F. Similarly, the G1F/G1F (G0F/G2F) peak shows partial separation. The first peak was assigned to G0F/G2F and G1F(1,3)/G1F(1,6) while the second peak likely represents G1F(1,6)/G1F(1,6). In addition, a third species [G1F(1,3)/G1F(1,3)] might populate the front of the peak. However, in this case peak asymmetry might provide an alternative explanation. For G1F/G2F, an early eluting peak [G1F(1,3)/G2F] was observed for the middle-up as well as the intact analysis. The affinity of G1F(1,6)/G2F was similar to G2F/G2F. A comparable glycoform ranking for mAb1 glycoforms is achieved in middle-up and intact FcɤRIIIa AC-MS analysis. The masses corresponded to the expected Fc fragments (Supplementary Table 2) and comparability of glycoform affinity ranking to the intact mAb1 was demonstrated by comparing retention time differences (Figure 3 and Supplementary Figure 4).

### FcɤRIIIa Affinity Analysis of Cetuximab Proteoforms

**Figure 4:**
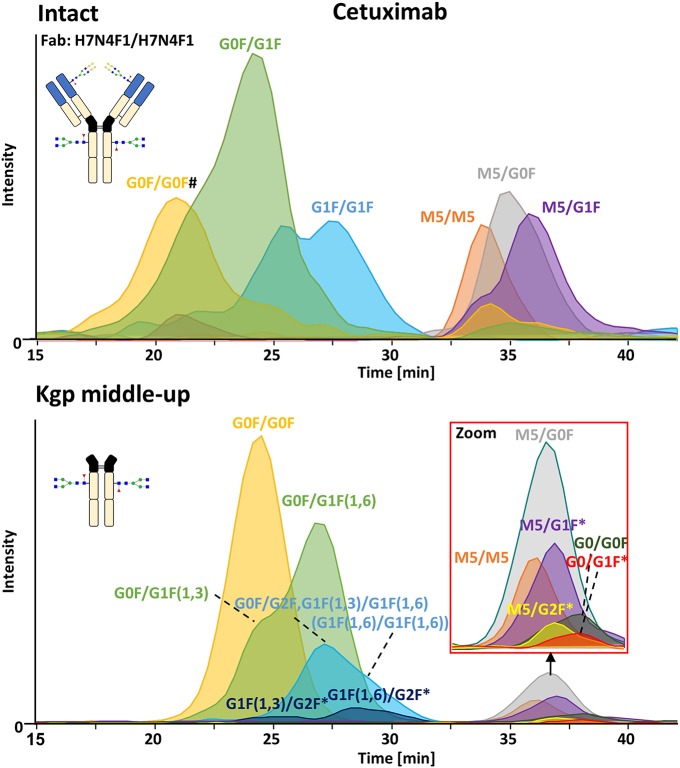
FcɤRIIIa AC-MS of cetuximab on intact and Kgp middle-up level. Intact analysis was restricted to the main Fab glycoform (H7N4F1/H7N4F1) and the most abundant Fc glycoforms (G0F, G1F, M5). G0F/G0F# is marked exemplarily for isomeric EICs: G0F/G1F exhibits the same EIC if the Fab glycoform is H7N4F1/H6N4F1. For middle-up FcɤRIIIa AC-MS analysis, EICs of all detected Fc glycoforms are presented. Data for C-terminal lysine variants (+K) are omitted for clarity. *indicates additional isomers listed in Supplementary Table 4.

**Figure 5:**
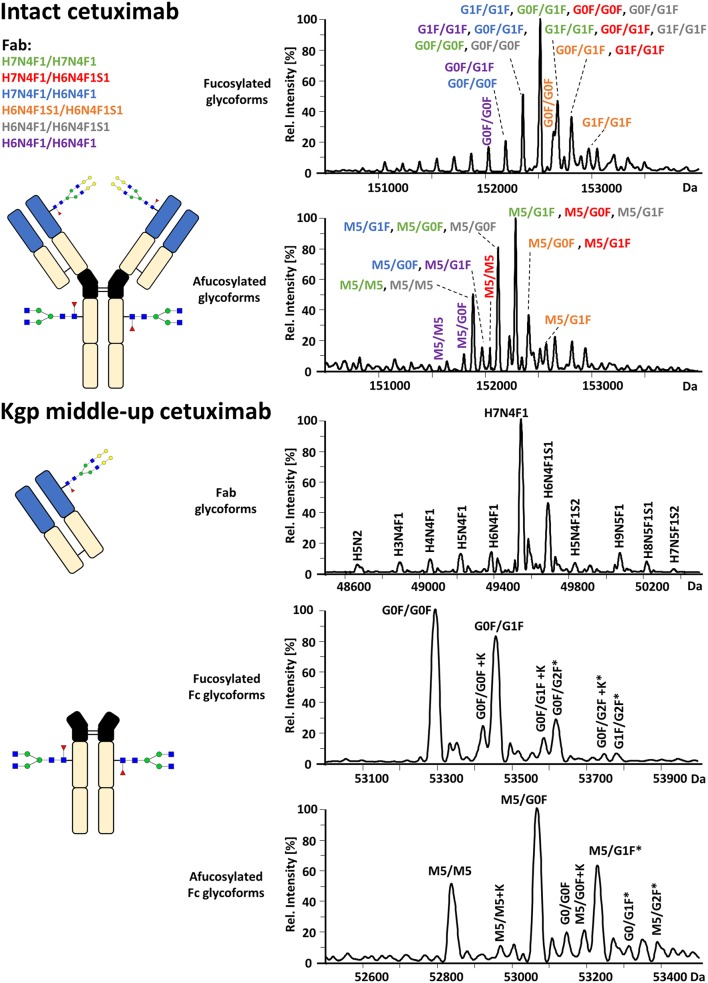
Assignment of cetuximab glycoforms on intact and Kgp middle-up level by FcɤRIIIa AC-MS. Fc glycoform assignment of intact cetuximab was restricted to 6 different Fab glycoform pairings indicated by a color code. The broad peaks often contain multiple isomeric and non-resolved glycoforms (Supplementary Table 3). Thirty four out of thirty six possibilities are assigned to peaks. For Kgp middle-up FcɤRIIIa AC-MS, the composition of Fab glycoforms were assigned as well as the Fc fragments (*indicates additional isomers). All observed deconvoluted masses for intact and middle-up analysis are listed in Supplementary Tables 3–5.

FcɤRIIIa AC-MS of intact cetuximab is illustrated in Figure 4 by EICs using *m/z* values of the three most abundant fucosylated and afucosylated Fc glycoforms, assuming H7N4F1/H7N4F1 as Fab glycosylation. Fab glycans are presented and discussed at a compositional level to avoid confusion with Fc glycans. Mainly fucosylated complex and high mannose glycoforms with little terminal galactose-α-1,3 galactose (α-gal) are described for the Fc glycosylation of SP2/0 produced cetuximab. For the Fab glycosylation, highly heterogeneous complex glycans with a high amount of α-gal and *N*-glycolylneuraminic acid (S) are reported (Jefferis, 2009; Biacchi et al., 2015). Intact cetuximab analysis proposed G0F/G1F and M5/G1F as the main fucosylated and (partially) afucosylated glycoforms, respectively (Figure 5). Based on literature, G0F/G0F and M5/G0F should be the most abundant Fc fucosylated and (partially) afucosylated glycoforms, respectively (Ayoub et al., 2013; Bern et al., 2018). This was later confirmed by middle-up analysis. FcɤRIIIa AC-MS of intact cetuximab reduced the MS spectral complexity by separating fucosylated from (partially) afucosylated Fc glycoforms (Figure 5) compared to previously reported intact analysis (Ayoub et al., 2013; Bern et al., 2018). However, one MS peak may still be assigned to various combinations of Fc and Fab glycoforms with the same mass or similar masses. The degree of overlapping glycoforms and the resulting assignment ambiguities were exemplified by permutating three high abundant Fab glycoforms (H6N4F1, H6N4F1S1, H7N4F1) with the three most abundant Fc glycoforms (G0F, G1F, M5) already resulting in 36 different combinations (Figure 5 and Supplementary Table 3). This is excluding heavy chain positional isomers within one site, such as G0F-H6N4F1/G1F-H7N4F1 and G0F-H7N4F1/G1F-H6N4F1. The theoretical number of possible glycoforms is significantly higher when considering all possible Fc and Fab glycans. Thus, at the intact level, assessing FcɤRIIIa affinity of cetuximab glycoforms by AC-MS based on EICs is prevented by the high number of isomeric and non-resolved proteoforms. For example, Fc glycoforms G0F/G0F and G0F/G1F will be extracted as the same mass, when combined with Fab glycans of composition H7N4F1/H7N4F1 and H7N4F1/H6N4F1, respectively. The MS analysis of mAbs is generally affected by an increased signal heterogeneity derived from additional non-resolved proteoforms and adducts (Campuzano et al., 2019). In particular for native MS of complex proteins, the applied deconvolution (algorithm, settings) can have an influence on the data evaluation with respect to resolving heterogeneous mass spectra (Campuzano et al., 2019). Cetuximab glycoforms, containing H7N4F1/H6N4F1S1 and H6N4F1/H6N4F1S1 Fab glycoform pairings (Figure 5 and Supplementary Table 3), were not resolved. A mass difference of 17 Da with H7N4F1/H7N4F1 and H6N4F1/H7N4F1 means the *m/*z difference for the most abundant charge state (28+) is 0.6 Th. This difference cannot be resolved under the applied conditions. Additionally, isomeric and non-resolved proteoforms lead to the distortion of the relative abundances as mentioned at the start of this paragraph. This becomes quite apparent when comparing the intact to the middle-up analysis in Figures 4, 5.

In contrast, middle-up FcɤRIIIa AC-MS of cetuximab simplified MS spectra enough to confidently assign Fc glycoforms and lysine variants (Figures 4, 5). Nonetheless, the Fc glycoform pairing assignments also showed some degree of ambiguity (Supplementary Table 4, e.g., M5/G1F vs. M6/G0F). However, these ambiguities were minor compared to the intact mass analysis of cetuximab. Relative abundancies were in line with literature on Fc/2 glycoforms (Ayoub et al., 2013; Bern et al., 2018). The Fc glycan pairing of cetuximab was so far only briefly mentioned in a recent study, using IdeS digestion and direct infusion with native MS conditions (Bern et al., 2018). We observed G0F/G0F as the main fucosylated glycoform. Additional galactosylation variants were observed as for mAb1. M5/G0F was determined as main (partially) afucosylated glycoform. Further high mannose glycoforms (M5/M5, M5/G1F, M5/G2F) were detected. Low amounts of G0/G0F, G0/G1F were also found. Relative FcɤRIIIa affinity was comparable to mAb1 (Figure 3) and/or consistent with literature (Lippold et al., 2019). For example, a strong decrease and a mild increase in FcɤRIIIa binding was observed for fucosylation and galactosylation, respectively (Dashivets et al., 2015; Thomann et al., 2015). High mannose glycoforms exhibited a higher affinity than the fucosylated glycoforms but their affinity is decreased compared to the afucosylated complex-type glycoforms (Yu et al., 2012). Interestingly, different pairings of high mannose glycoforms with fucosylated complex-type glycans (M5/G1F. M5/G2F) could be studied and were found with slightly higher FcɤRIIIa affinity compared to M5/M5. M5/G1F and M5/G2F showed a slightly increased affinity over M5/G0F. This confirms and extends our previous findings, which were limited to the comparison of low abundant M5/M5 and M5/G0F (Lippold et al., 2019). Though it is difficult to compare affinity of the intact and the Fc, the latter seems to show a higher affinity (Figure 4). This is comparable to the negative influence of the Fab on FcRn binding reported for cetuximab (Schlothauer et al., 2013).

Furthermore, C-terminal lysine variants (+K, Figure 1) could be studied in the middle-up analysis with respect to FcɤRIIIa affinity. Investigations of incomplete lysine clipping with respect to different Fc glycoforms and FcɤRIIIa affinity have not been described yet (Brorson and Jia, 2014). The C-terminal lysine appeared not to influence the Fc glycoform retention strongly (Supplementary Table 4) which is not surprising as the receptor binds far away from the C-terminus (Figure 1). In contrast, C-terminal lysine may interfere with complement activation. However physiological relevance may anyhow be small as the lysine is enzymatically removed upon administration (van den Bremer et al., 2015).

Moreover, middle-up FcɤRIIIa AC-MS allowed a simultaneous determination of Fab glycosylation by evaluating the injection peak (Figure 5 and Supplementary Table 5). Fab glycosylation might be relevant for antigen binding and pharmacokinetic behavior (Huang et al., 2006; Jefferis, 2007). In total, 11 Fab glycoforms were assigned. Our results are qualitatively and quantitatively in line with reported Fab glycosylation of SP2/0 produced cetuximab (Ayoub et al., 2013; Bern et al., 2018). H7N4F1 was determined to be the most abundant glycoform followed by H6N4F1S1, in line with a previous report (Ayoub et al., 2013) and in contrast to a recent study (H7N4F2) (Bern et al., 2018). The two differently reported Fab glycoforms vary only by 1 Da. However, based on in-depth structural studies on cetuximab glycosylation reporting a high amount of S and only minor abundancies of antenna fucosylation, Fab glycoforms are more likely to contain H6N4F1S1 than additional H7N4F2 (Wiegandt and Meyer, 2014).

By applying middle-up FcɤRIIIa AC-MS to cetuximab, 10 Fc glycoforms could be assigned, of which 5 Fc glycoforms were also found with the C-terminal lysine (amounting to 21 possible isomers, Supplementary Table 4). The combination of the 21 assigned Fc glycoform pairings (Supplementary Table 4) with the 11 assigned Fab glycoforms (Supplementary Table 5), belonging to 66 Fab glycoform pairings in theory, would result in 1,386 proteoforms for the intact cetuximab. This underlines the limitations of intact FcɤRIIIa AC-MS for Fab glycosylated IgGs and highlights the degree of simplification obtained by the middle-up workflow.

## Conclusion

IgG Fc, generated by three established IgG middle-up proteases (IdeS, SpeB, Kgp), demonstrated differences in retention behavior in FcɤRIIIa AC-MS. Kgp derived Fc showed a remarkably similar retention profile and glycoform ranking as the intact mAb. Advantages of the Kgp middle-up FcɤRIIIa AC-MS workflow were demonstrated in the application to the Fab glycosylated therapeutic mAb, cetuximab. The middle-up workflow provided significantly reduced MS complexity compared to the intact level. Consequently, it revealed important information about cetuximab Fc glycoforms by enabling their confident assignment and quantitation while retaining the Fc pairing and FcɤRIIIa AC retention. Simultaneously, Fab glycosylation could be determined.

In the future, middle-up AC-MS might also be a tool to investigate the interplay of the Fab and the Fc in structure-function studies of IgG-Fc receptor interactions. Since the presented workflow with Kgp is limited to human IgG1, further development of hinge-specific antibody proteases with broader subclass coverage would be highly desired. AC-MS workflows may thus be further expanded toward clinical applications and polyclonal therapeutic samples (e.g., intravenous IgGs). Moreover, the complexity of antibody-derived therapeutics is growing. Hence, the interest in middle-up approaches might further evolve in order to characterize new generations of therapeutic proteins.

## Data Availability Statement

The raw data supporting the conclusions of this manuscript will be made available by the authors, without undue reservation, to any qualified researcher.

## Author Contributions

SL performed and evaluated the experiments, supported by SN and supervised by DF. SL, MW, and DF designed the experiments. SL, SN, and DF drafted the manuscript. All authors contributed to finalizing the manuscript.

### Conflict of Interest

The authors declare that the research was conducted in the absence of any commercial or financial relationships that could be construed as a potential conflict of interest.
