# Supplementary material for: Proteoform-Resolved FcɤRIIIa Binding Assay for Fab Glycosylated Monoclonal Antibodies Achieved by Affinity Chromatography Mass Spectrometry of Fc Moieties
Source: Front Chem. 2019 Oct 24;7:698. doi: 10.3389/fchem.2019.00698 (PMC6822288; doi:10.3389/fchem.2019.00698)
Supplement: Supplementary file 1 [file Table_1.docx]

Supplementary Material

**
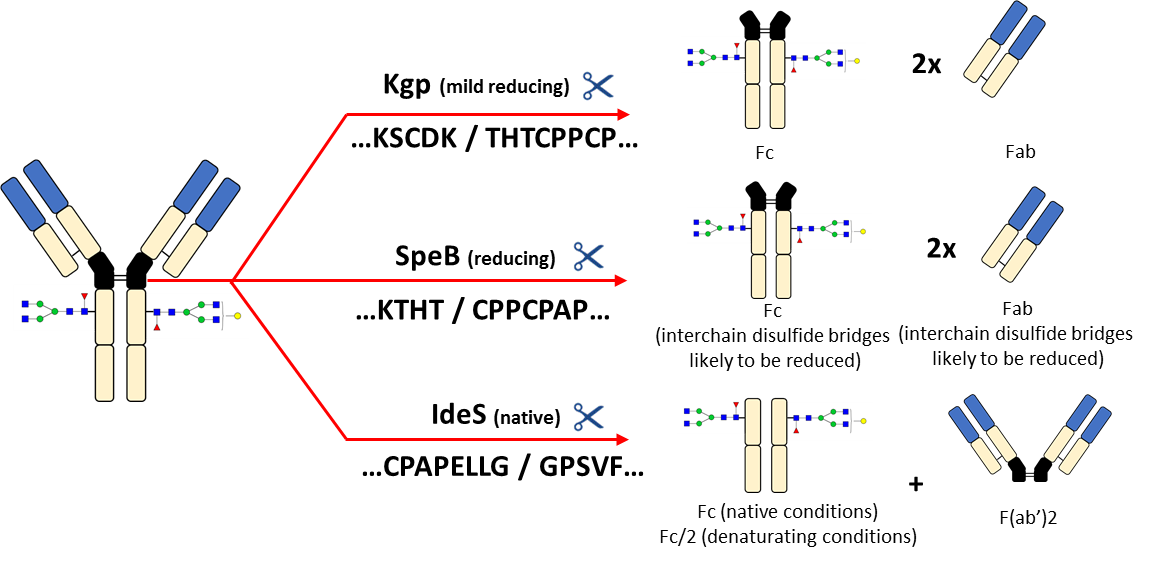
**

**Supplementary Figure 1: Overview of cleavage sites, conditions and products of tested IgG proteases, Kgp, SpeB and IdeS.** IdeS works without reducing conditions (100 mM ammonium bicarbonate pH 7 was applied in this study), Kgp under mild reducing conditions (100 mM Tris pH8, 2 mM cysteine was applied in this study) and SpeB under strong reducing conditions (100 mM Tris pH8, 1 mM DTT was applied in this study). Interchain disulfide bridges for Kgp digestion are expected to be intact (Moelleken et al., 2017). For SpeB digestion, interchain disulfide bridges are likely to be reduced (Sjögren et al., 2017).

**Supplementary Table 1: Overview of glycoforms described in this study.**

| **Nomenclature** | **Composition*** | **Structure**** | **Fc/Fab** |
| --- | --- | --- | --- |
| G0F | H3N4F1 | 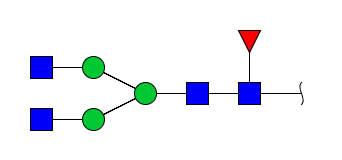 | Fc, Fab |
| G1F  G1F(1,3)  G1F(1,6) | H4N4F1 | 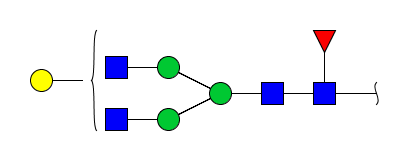 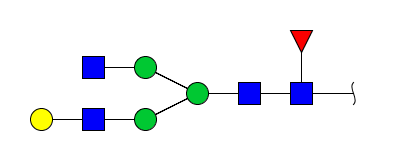 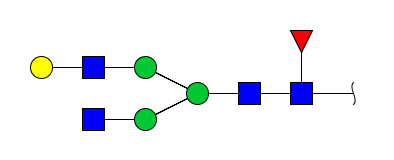 | Fc, Fab |
| M5 | H5N2 | 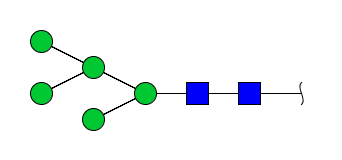 | Fc, Fab |
| G2F | H5N4F1 | 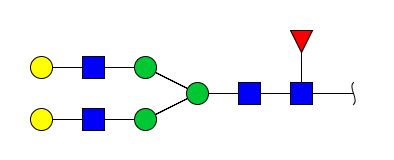 | Fc, Fab |
| M6 | H6N2 | 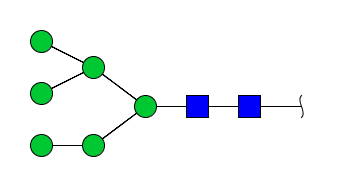 | Fc |
| G2FαGal1 | H6N4F1 | 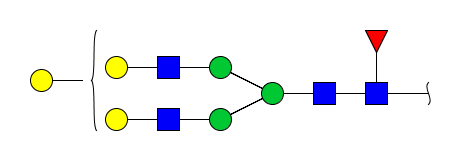 | Fc, Fab |
| G0 | H3N4 | 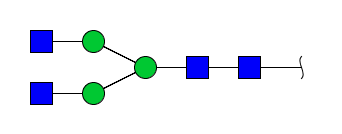 | Fc |
| G1 | H4N4 | 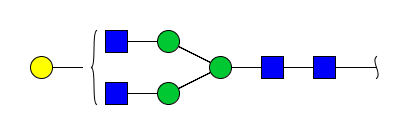 | Fc |
| G2FαGal2 | H7N4F1 | 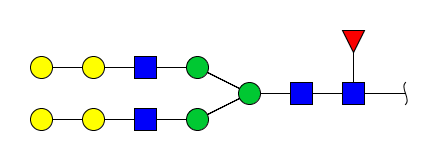 | Fc, Fab |
| G2FαGal1S1 | H6N4F1S1 | 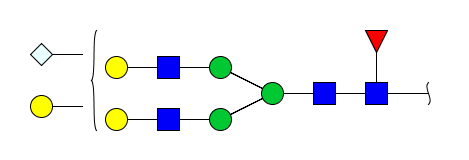 | Fc, Fab |
| H5N4F1S2 | | 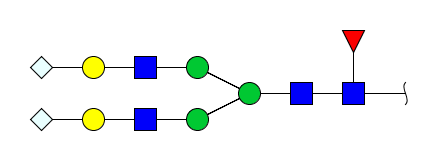 | Fab |

**Supplementary Table 1 continued**

| **Nomenclature** | **Composition*** | **Structure**** | **Fc/Fab** |
| --- | --- | --- | --- |
| H9N5F1 | | 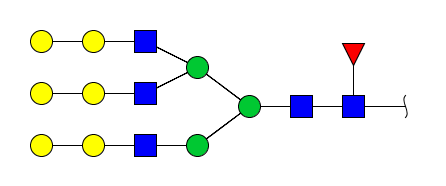 | Fab |
| H8N5F1S1 | | 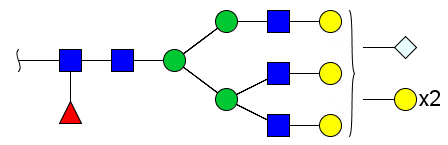 | Fab |
| H7N5F1S2 | | 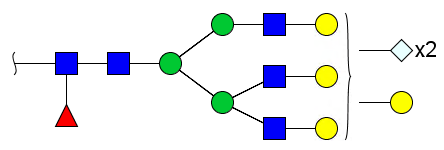 | Fab |

* H = hexose, N = *N*-acetylhexosamine, F = fucose, S = *N*-glycolylneuraminic acid

** As expected for the Fc and Fab glycosylation of CHO and SP2/0 produced mAbs


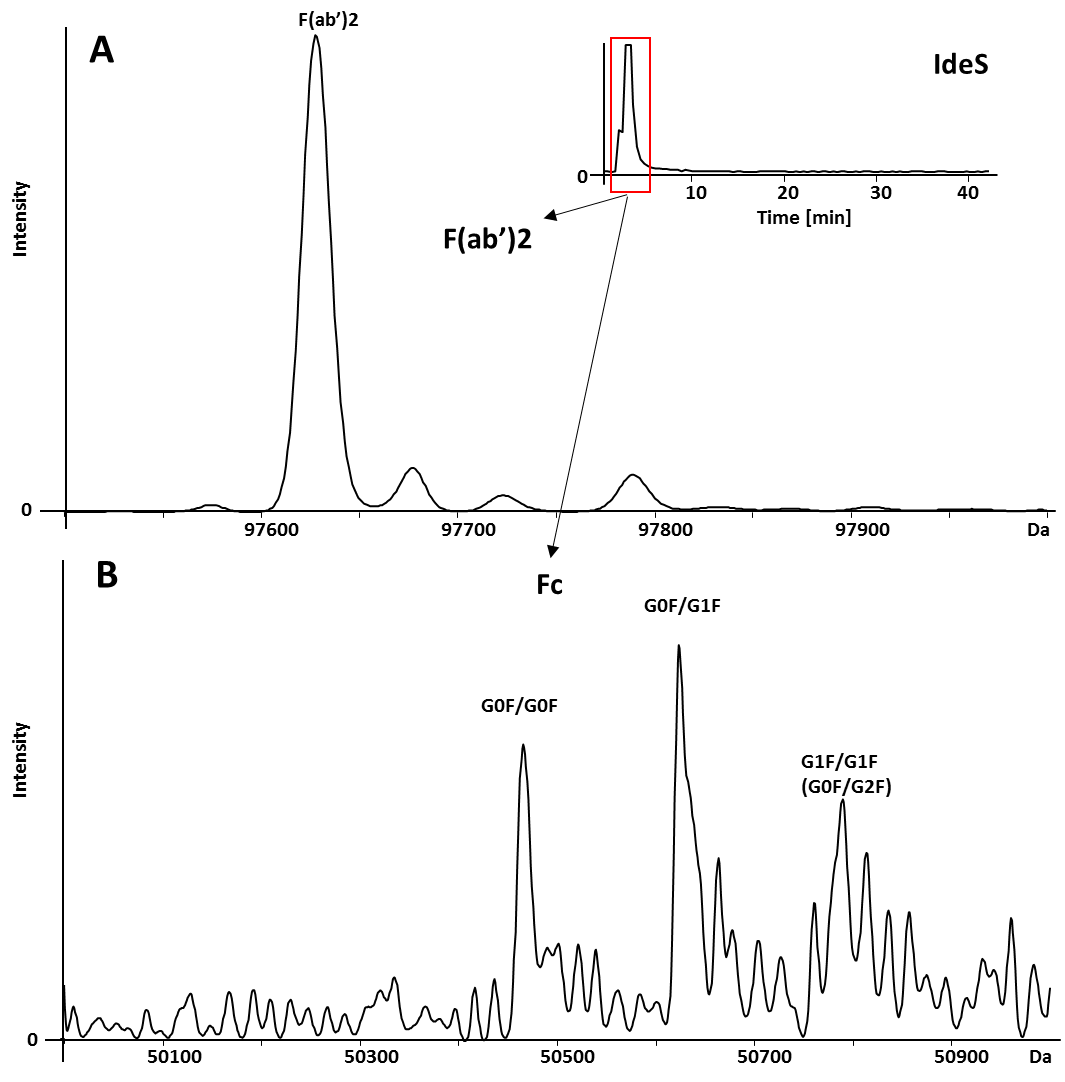


**Supplementary Figure 2:** **Deconvoluted mass spectra of the injection peak for the** **FcɣRIIIa AC-MS analysis of Ides digested mAb1.** (A) The F(ab’)2 (97500 Da to 98000 Da) was observed with high signal intensity. (B) The intact Fc (50000 Da to 51000 Da**)** was detected under the applied native MS conditions as shown for the three main glycoforms. Signal intensity for co-eluting Fc was very low compared to the Fab part.


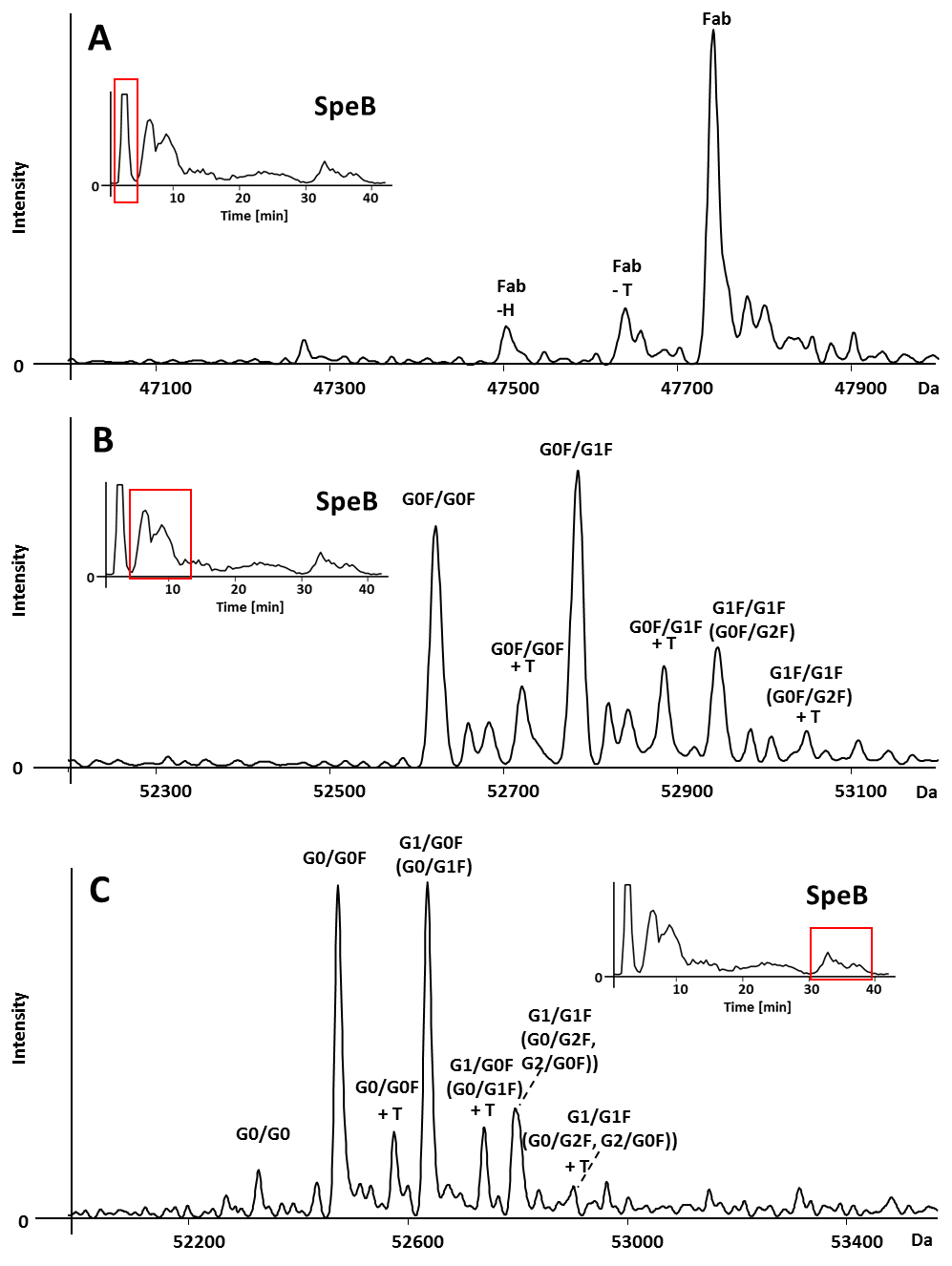


**Supplementary Figure 3:** **Deconvoluted mass spectra for SpeB digestion products of mAb1 in FcɣRIIIa AC-MS.** (A) Injection peak showing the Fab (45000 Da to 50000 Da) with T and H losses. (B) Main 2x fucosylated Fc glycoforms (50000 Da to 55000 Da) observed from 5 min to 13 min and (C) main afucosylated Fc glycoforms (50000 Da to 55000 Da) observed from 32 min to 40 min with high retention. Unspecific cleavages of SpeB were also observed for Fc (+ T). Fc with an additional H was not observed.

**Supplementary Table 2:** **Overview of major Fc glycoforms observed in deconvoluted mass spectra of Kgp middle-up FcɣRIIIa AC-MS of mAb1 (50000 Da to 55000 Da).**

| **Nomenclature** | **Theoretical mass (Da)** | **Deconvoluted mass (Da)** | **Delta (Da)** | **Retention time (min)** |
| --- | --- | --- | --- | --- |
| G0F/G0F | 53295.2 | 53295.5 | 0.3 | 25.1 |
| G0F/G1F(1,3) | 53457.4 | 53457.9 | 0.5 | 25.3 |
| G0F/G1F(1,6) |  |  |  | 27.6 |
| G0F/G2F, G1F(1,3)/G1F(1,6) | 53619.5 | 53620.0 | 0.5 | 27.9 |
| G1F(1,6)/G1F(1,6) |  |  |  | 29.7 |
| G1F(1,3)/G2F | 53781.7 | 53782.3 | 0.6 | 27.6 |
| G1F(1,6)/G2F |  |  |  | 30.1 |
| G2F/G2F | 53943.8 | 53945.0 | 1.2 | 30.2 |
| G0/G0F | 53149.1 | 53148.7 | -0.4 | 38.1 |
| G1/G0F (G0/G1F) | 53311.2 | 53311.6 | 0.4 | 38.4 |
| G1/G1F (G0/G2F, G2/G0F) | 53473.4 | 53473.0 | -0.4 | 38.8 |
| G0/G0 | 53003.0 | 53003.4 | 0.4 | 39.1 |


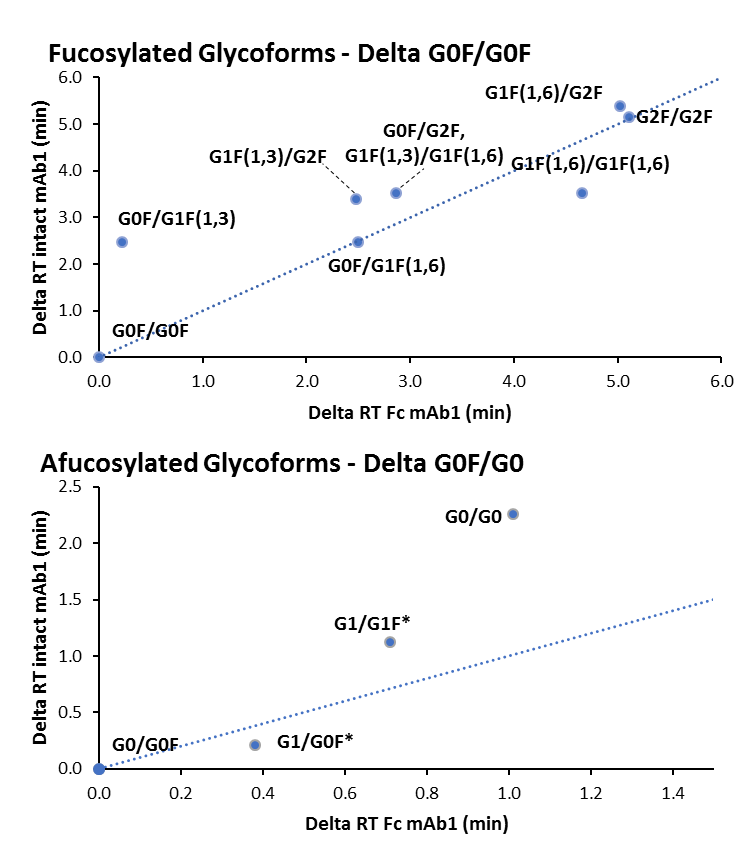


**Supplementary Figure 4:** **Retention time correlation of intact mAb1 and Kgp derived Fc glycoforms of mAb1.** Retention time was correlated by calculating differences of fucosylated glycoforms to the G0F/G0F glycoform (upper panel) and differences of afucosylated glycoforms to the G0/G0F glycoform (lower panel). The blue dotted line represents a theoretical correlation line. Asterisks indicate multiple possibilities for glycoform assignment (**Supplementary Table 2**). For fucosylated glycoforms, partially separated peaks of Kgp derived Fc and intact mAb1 were considered. For Fc glycoforms, slightly smaller differences were observed for the afucosylated glycoforms. However, the tendencies of different glycoforms were overall consistent. High affinity glycoforms of mAb1 (G1/G1F* and G0/G0) showed increased tailing and distorted retention time behavior as compared to the previously reported analysis of mAb1 (Lippold et al., 2019). That might indicate a potentially changed column performance for high affinity glycoforms.

**Supplementary Table 3:** **Theoretical and deconvoluted intact masses of cetuximab (145000 Da to 155000 Da) permutated with H7N4F1, H6N4F1, H6N4F1S1 (Fab glycoforms) and G0F, G1F, M5 (Fc glycoforms).**

| **Fab** | **Fc** | **Theoretical (Da)** | **Deconvoluted * (Da)** | **Difference (Da)** |
| --- | --- | --- | --- | --- |
| **H7N4F1/H7N4F1** | **G0F/G0F** | 152351.7 | 152352.7 (F3) | 1.0 |
|  | **G0F/G1F** | 152513.8 | 152512.9 (F4) | -0.9 |
|  | **M5/G0F** | 152123.4 | 152125.7 (AF6) | 2.3 |
|  | **G1F/G1F** | 152676.0 | 152675.7 (F6) | -0.3 |
|  | **M5/G1F** | 152285.6 | 152289.9 (AF7) | 4.3 |
|  | **M5/M5** | 151895.2 | 151889.8 (AF3) | -5.4 |
| **H7N4F1/H6N4F1S1** | **G0F/G0F** | 152496.8 | F4 | 16.1 (17 expected) |
|  | **G0F/G1F** | 152658.9 | F6 | 16.8 (17 expected) |
|  | **M5/G0F** | 152268.6 | AF7 | 21.3 (17 expected) |
|  | **G1F/G1F** | 152821.1 | F7 | -15.4 (-17 expected) |
|  | **M5/G1F** | 152430.7 | AF8 | -19.4 (-17 expected) |
|  | **M5/M5** | 152040.3 | 152049.1 (AF5) | 8.8 |
| **H7N4F1/H6N4F1** | **G0F/G0F** | 152189.6 | 152190.9 (F2) | 1.3 |
|  | **G0F/G1F** | 152351.7 | 152352.7 (F3) | 1.0 |
|  | **M5/G0F** | 151961.3 | AF4 | 12.8 (not assigned) |
|  | **G1F/G1F** | 152513.8 | 152512.9 (F4) | -0.9 |
|  | **M5/G1F** | 152123.4 | 152125.7 (AF6) | 2.3 |
|  | **M5/M5** | 151733.1 | n.d. | - |
| **H6N4F1S1/H6N4F1S1** | **G0F/G0F** | 152641.9 | 152639.9 (F5) | -2.0 |
|  | **G0F/G1F** | 152804.1 | 152805.7 (F7) | 1.6 |
|  | **M5/G0F** | 152413.7 | 152411.3 (AF8) | -2.4 |
|  | **G1F/G1F** | 152966.2 | 152967.1 (F8) | 0.9 |
|  | **M5/G1F** | 152575.8 | 152576.6 (AF9) | 0.8 |
|  | **M5/M5** | 152185.4 | n.d. | - |
| **H6N4F1/H6N4F1S1** | **G0F/G0F** | 152334.7 | F3 | 18.0 (17 expected) |
|  | **G0F/G1F** | 152496.8 | F4 | 16.1 (17 expected) |
|  | **M5/G0F** | 152106.4 | AF6 | 19.3 (17 expected) |
|  | **G1F/G1F** | 152658.9 | F6 | 16.8 (-17 expected) |
|  | **M5/G1F** | 152268.6 | AF7 | 21.3 (17 expected) |
|  | **M5/M5** | 151878.2 | AF3 | 11.6 (17 expected) |
| **H6N4F1/H6N4F1** | **G0F/G0F** | 152027.4 | 152029.4 (F1) | 2.0 |
|  | **G0F/G1F** | 152189.6 | 152190.9 (F2) | 1.3 |
|  | **M5/G0F** | 151799.2 | 151804.1 (F2) | 4.9 |
|  | **G1F/G1F** | 152351.7 | 152352.7 (F3) | 1.0 |
|  | **M5/G1F** | 151961.3 | AF4 | 12.8 (not assigned) |
|  | **M5/M5** | 151570.9 | 151574.0 (AF1) | 3.1 |

*** Non-resolved glycoforms are assigned to the closest mass (indicated as Fx or AFx). F= Fucosylated glycoforms, AF = Afucosylated glycoforms, x = number of assigned peak).**

**Supplementary Table 4:** **Fc glycoforms for cetuximab by Kgp middle-up FcɣRIIIa AC-MS (Fc: 50000 Da to 55000 Da).**

| **Nomenclature** | **Theoretical mass (Da)** | **Deconvoluted mass (Da)** | **Delta (Da)** | **Relative area (%)** | **Retention time (min)** |
| --- | --- | --- | --- | --- | --- |
| G0F/G0F | 53295.2 | 53295.6 | 0.4 | 32.5 | 24.4 |
| G0F/G1F(1,3) | 53457.4 | 53457.9 | 0.5 | 9.0 | 25.3 |
| G0F/G1F(1,6) |  |  |  | 22.1 | 27.0 |
| G1F/G1F (G0F/G2F) | 53619.5 | 53619.0 | -0.5 | 11.2 | 27.3 |
| G1F(1,3)/G2F (G0F/G2FαGal1) | 53781.7 | 53781.0 | -0.7 | 0.9 | 25.7 |
| G1F(1,6)/G2F (G0F/G2FαGal1) |  |  |  | 1.9 | 28.5 |
| G0F/G0F +K | 53423.4 | 53423.6 | 0.2 | 4.7 | 24.7 |
| G0F/G1F +K | 53585.6 | 53587.0 | 1.4 | 3.9 | 27.2 |
| G1F/G1F (G0F/G2F) +K | 53747.7 | 53748.8 | 1.1 | 1.2 | 27.5 |
| M5/M5 | 52838.8 | 52837.1 | -1.7 | 2.0 | 36.1 |
| M5/G0F | 53067.0 | 53066.6 | -0.4 | 5.4 | 36.6 |
| M5/G1F (M6/G0F) | 53229.1 | 53228.4 | -0.7 | 2.6 | 36.9 |
| M5/G2F (M6/G1F) | 53391.3 | 53389.3 | -2.0 | 0.6 | 37.0 |
| M5/M5 +K | 52966.9 | 52969.0 | 2.1 | 0.3 | 35.9 |
| M5/G0F +K | 53195.2 | 53193.7 | -1.5 | 0.6 | 36.1 |
| G0/G0F | 53149.1 | 53146.2 | -2.9 | 1.0 | 37.9 |
| G0/G1F (G1/G0F) | 53311.2 | 53312.7 | 1.5 | 0.4 | 37.9 |

**Supplementary Table 5:** **Cetuximab Fab glycoforms by Kgp middle-up FcɣRIIIa AC-MS (Fab: 47000 Da to 52000 Da).**

| **Composition** | **Theoretical mass (Da)** | **Deconvoluted mass (Da)** | **Delta (Da)** | **Relative area (%)** |
| --- | --- | --- | --- | --- |
| H5N2 | 48669.4 | 48669.5 | 0.1 | 2.4 |
| H3N4F1 | 48897.7 | 48896.5 | -1.2 | 2.6 |
| H4N4F1 | 49059.8 | 49058.8 | -1.0 | 3.1 |
| H5N4F1 | 49222.0 | 49222.2 | 0.2 | 4.9 |
| H6N4F1 | 49384.1 | 49385.8 | 1.7 | 4.5 |
| H7N4F1 | 49546.2 | 49545.4 | -0.8 | 49.1 |
| H6N4F1S1 | 49691.4 | 49690.5 | -0.9 | 22.8 |
| H5N4F1S2 | 49836.5 | 49834.6 | -1.9 | 1.4 |
| H9N5F1 | 50073.7 | 50075.3 | 1.6 | 5.7 |
| H8N5F1S1 | 50218.8 | 50218.6 | -0.2 | 2.8 |
| H7N5F1S2 | 50363.9 | 50363.8 | -0.1 | 0.7 |

**References**

Lippold, S., Nicolardi, S., Domínguez-Vega, E., Heidenreich, A.-K., Vidarsson, G., Reusch, D., et al. (2019). Glycoform-resolved FcɣRIIIa affinity chromatography–mass spectrometry. *mAbs***,** in press. doi: 10.1080/19420862.2019.1636602.

Moelleken, J., Endesfelder, M., Gassner, C., Lingke, S., Tomaschek, S., Tyshchuk, O., et al. (2017). GingisKHAN™ protease cleavage allows a high-throughput antibody to Fab conversion enabling direct functional assessment during lead identification of human monoclonal and bispecific IgG1 antibodies. *mAbs* 9(7)**,** 1076-1087. doi: 10.1080/19420862.2017.1364325.

Sjögren, J., Andersson, L., Mejàre, M., and Olsson, F. (2017). "Generating and Purifying Fab Fragments from Human and Mouse IgG Using the Bacterial Enzymes IdeS, SpeB and Kgp," in *Bacterial Pathogenesis: Methods and Protocols,* eds. P. Nordenfelt & M. Collin. (New York, NY: Springer New York), 319-329.
